# Conceptual Study and Manufacturing of a Configurable and Weld-Free Lattice Base for Automatic Food Machines

**DOI:** 10.3390/ma14071692

**Published:** 2021-03-30

**Authors:** Alessandro Pirondi, Andrea Liberini, Flavio Rocchi

**Affiliations:** 1 Department of Engineering and Architecture, University of Parma, Parco Area delle Scienze 181/A, 43124 Parma, Italy; ing.liberini@gmail.com; 2 Promec SrL, Strada Fornace 4, 43010 Bianconese, Italy; direzione@promec-srl.com

**Keywords:** conceptual design, lattice structure, food machines

## Abstract

The study is aimed at developing a modular lattice base for automatic food machines, starting with a solution already patented by some of the authors. In this case, welded carpentry modules were interlocked with a system of profiles and metal inserts, also in welded carpentry, and the union was stabilized by structural adhesive bonding. Since welding involves long processing times and thermal distortions to be restored later, the driver of this study is to limit the use of welding as much as possible while increasing the modularity of the construction. For this purpose, various solution concepts have been generated where a common feature is the presence of rods of the same geometry and section to be joined together in configurable structural nodes. The concepts are qualitatively evaluated in light of the requirements, and the selected concept is digitally and physically prototyped. The prototype has been in service from over 5 years without showing any problems whatsoever.

## 1. Introduction

**Figure 1:**
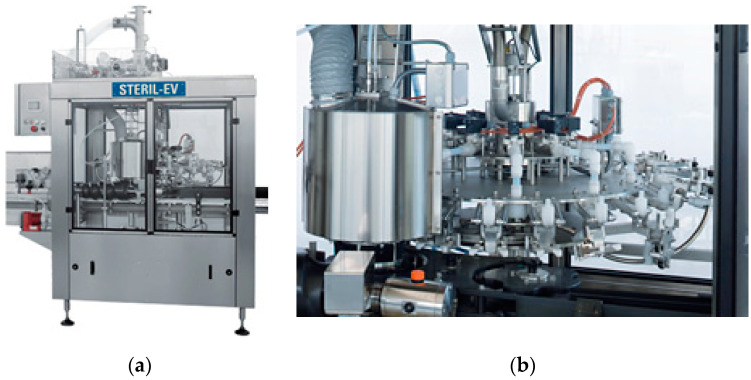
Example of a sterilizing machine for a bottling line: complete machine (**a**); rotating carousel (**b**). From www.promec-srl.com (accessed on 18 February 2021).

Food packaging machines need to follow market trends, customer needs, technological improvements, standards, and regulations, which differ from other types of packaging machines [1,2,3], and for this reason, the design and construction of their structure must be as reconfigurable as possible and characterized by few elements. The machines referred to in this study are used in food beverage bottling lines. The base constitutes the fixed structural part of these machines, while the filling operation is accomplished by modules mounted on the periphery of a rotating part called carousel (Figure 1). The working environment requires the construction to be entirely in stainless steel, whose cost is considerably higher than that of the common unalloyed steel. The loads on the base are given by the suspended mass, the drive torque given by the motorization, and the inertial forces related to the movements impressed on the bottle. The optimization of the relationship between stiffness and mass of the base is essential to contain the quantity of material used and the relative cost, while keeping a good structural performance. The construction of an optimized base must also limit the use of welded carpentry, which involves long processing times and thermal distortions to be restored later. The TIG (tungsten inert gas) or MIG (metal inert gas) welding of stainless steel is not troublesome for austenitic stainless steel grades, provided that the use of a weld metal containing a small amount of ferrite (e.g., welding AISI 304 using AISI 308 weld metal) to avoid shrinkage cracks in HAZ (heat-affected zone). Different stainless steel grades can also be laser-welded with some precautions, obtaining a good-quality joint as shown in [4,5]. However, according to the guidelines [6] on hygienic design of weldments for food machines and plant applications, it is also necessary to etch and passivate the weld seam, with a further increase in time and cost. Furthermore, for safety reasons, during welding there can be no other personnel within the same assembly station, making it impossible to carry out other operations in parallel with welding.

The design process of a new product, called product development process (PDP), is divided into three phases: (i) conceptual phase, (ii) system design phase, and (iii) detail phase [7]. In the conceptual phase, various methods and tools are available to help designers in defining a correct product architecture. The product architecture is a flowchart that describes how the product functions are gathered to create modules and how the modules interact with each other [8], and most of the choices made in the conceptual phase have an impact on the overall design of the product [7].

To identify a product architecture, it is necessary to map all the functions of the product and then collect these functions into groups (i.e., modules). There are several tools available to perform functional map derivation, such as function–means tree [9], functional evolution process (FEP) [10], and functional analysis [7]. The creation of modules is a complex activity: in the literature, there are several methods to perform modularization, (i.e., obtain a modular product with respect to a specific purpose (for example, reduce the number of elements of the product and improve customization and/or product maintenance). Some methods use an objective function that has to be minimized [11,12]; other methods use heuristics to derive optimal modules [13]. Regarding the food industry, there are several studies on improving food packaging processes. Some studies focus on the environmental impact through a life-cycle assessment (LCA) [14]. Others have sought to improve the automation of food packaging machines [15,16,17]. Open innovation methods have also been proposed to improve the food packaging sector [18,19]. However, from a design point of view, although the methods described above are well known in the literature and have been used in many fields (e.g., automotive, household appliances, and electronic boards), their use in food industry machines, in particular in the development of packaging systems, is still rarely applied [20,21].

The novelty that this study aims to introduce is therefore twofold: from the point of view of automatic food machines, to develop a modular lattice base that is weld-free, is optimized in strength and stiffness with respect to weight, and constitutes a breakthrough innovation for the design of such components in this field; from the methodological point of view, to operate the development according to a structured PDP design approach, such as that described in [7], which is also uncommon in the field of automatic food machines.

## 2. Materials and Methods

### 2.1. Materials Selection

When selecting materials for the construction of food machines, it should be borne in mind that they must comply with the standards of “hygienic” design [6,22,23]. In particular, concerning the materials in contact with food, the following documents constitute the references for metals and plastics:

- Italian Decree of Ministry of 21 March 1973, with subsequent amendments, regulates packaging, containers, and tools intended to come into contact with food substances or substances for personal use;
- Italian Decree of Ministry of 27 October 2009, n. 176, for stainless steels;
- Italian Decree of Ministry of 21 December 2010, n. 258, for stainless steels;
- Regulation (EC) No. 1935/2004 of the European Parliament and of the Council of 27 October 2004 on materials and articles intended to come into contact with food;
- Commission Regulation (EU) No. 10/2011 of 14 January 2011 on plastic materials and articles intended to come into contact with food;
- EHEDG (European Hygienic Engineering and Design Group) doc. 32—materials of construction for equipment in contact with food.

European regulations and the EHEDG guidelines refer to positive lists of materials that either are included in the regulations themselves (such as in the 10/2011 for plastics) or can be found in national decrees of countries belonging to the European Union. Therefore, the Italian Decree of Ministry of 21 March 1973, delimitates the perimeter of materials that can be used in equipment in contact with food. As for metals, the enclosed list contains essentially stainless steels of austenitic and ferritic grades, where austenitic should be preferred where problems of corrosion due to organic substances, chlorides, sulfides, and so forth, are stronger; that is where the part is in direct contact with food. Therefore, the carousels of a beverage filling machines are entirely made of AISI 304 or 316 austenitic stainless steel (low-carbon grade if to be welded). Even though the base is not in direct contact with food, it is subjected to daily/weekly external cleaning operations that consist in this sequence: washing with alkaline solution, rinsing with water, washing with acid solution, and rinsing with water. In order to avoid corrosion due to these strong alkaline and acid solutions, also the base should be built using austenitic stainless steel that is at least of AISI 304 grade. The latter has therefore been chosen for the study.

Regarding polymers, the weld-free construction of the base foresees the use of a structural adhesive in some if not all the connections. Since the base is not in direct contact with food, there is no potential risk of migration of substances from the adhesive to the product; therefore, a food-grade characteristic is not necessary. The adhesive is selected based on the lap strength on stainless steel, the resistance to the chemicals used in cleaning and sanitization, a fixture time that is reasonably long to perform the assembly without problems, and a curing time acceptable in comparison with the overall machine assembly time. Cold bonding and the supply of a cartridge package are further requirements. The choice falls on Loctite EA 9466 (Henkel Italia, Milan, Italy), a two-component cartridge-disposable epoxy adhesive that fixes in 3 h and cures completely in 24 h at room temperature. The basic mechanical properties according to the supplier datasheet are shown in Table 1. The surface preparation for bonding includes degreasing, sandpapering with emery paper, and cleaning with Loctite 7063 cleaner (Henkel Italia, Milan, Italy).

Single-lap adhesive joints of AISI 304 L were tested in [24] using Loctite EA 9466 after aging in alkaline (3%–10% KOH) or acid (30%–50% H_3_PO_4_ or a mixture of 1%–3% peracetic acid +3%–10% acetic acid +10%–20% hydrogen peroxide) solutions. The lap shear strength has shown a minimum value of 10.6 MPa, which is about 56.8% of the unaged strength (the latter obtained on a mirrorlike surface finish just decreased and cleaned), which is anyway a good value if the adhesive is used mainly to give stability to a form connection likewise [21]. Some spontaneous debondings were recorded in the alkaline solution, but they can also be attributed to the very hard aging condition applied in the lab (continuous immersion in the solution heated up to 40 °C), while in reality, the treatment is daily/weekly and the chemicals are rinsed with water afterwards.

### 2.2. PDP Methodology

**Figure 2:**
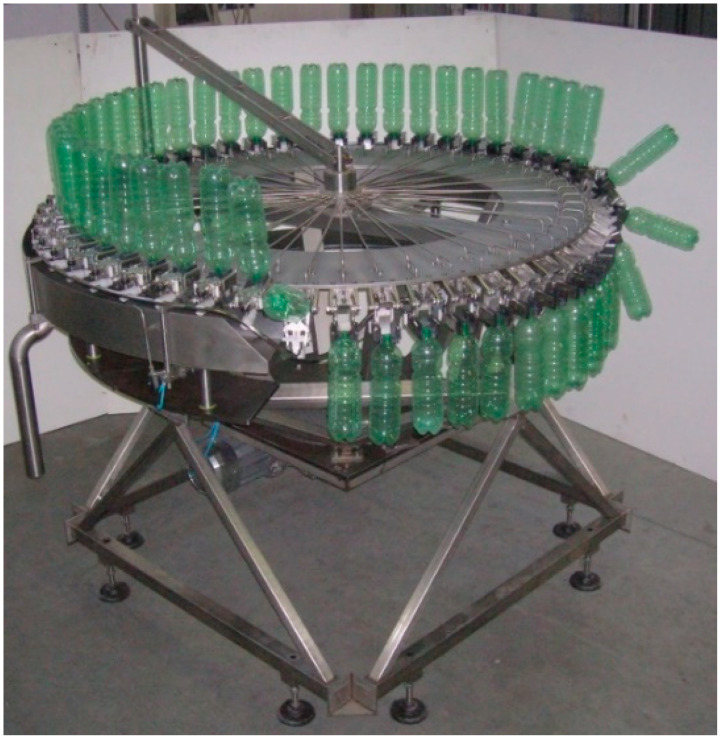
Bottle sterilizer machine with the base developed in [21]. External protection is not present in order to display the base.

**Figure 3:**
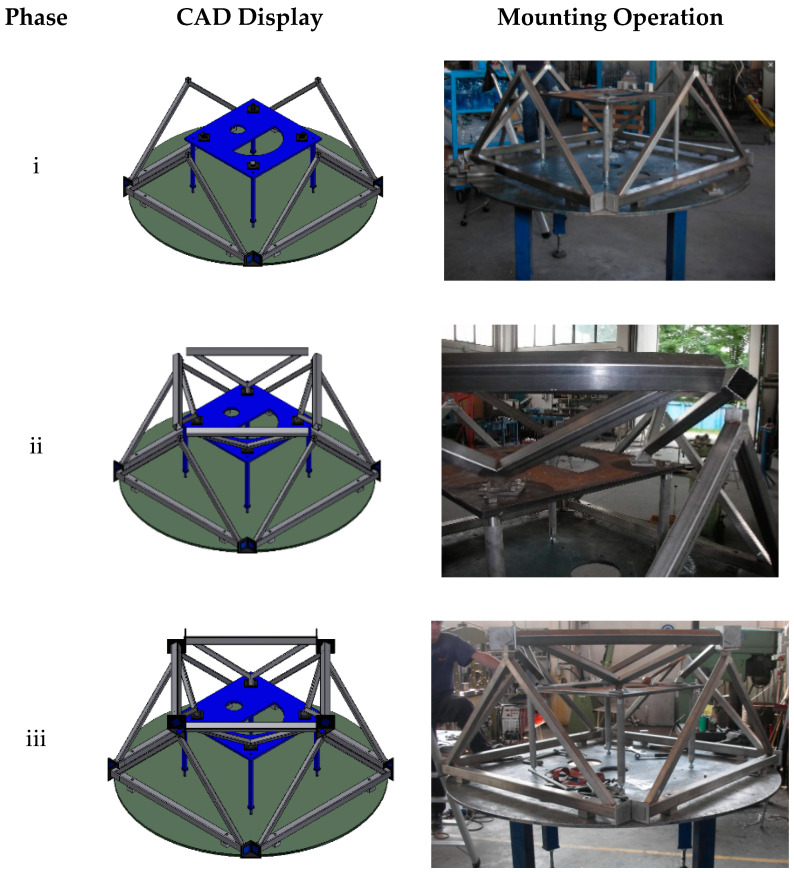
Steps of the assembly of the [21] base: (i) joining of larger modules; (ii) mounting of the smaller modules onto the reference (in blue); (iii) joining of larger and smaller modules.

The modular lattice base concept developed, prototyped, and patented by some of the authors for a sterilizing machine [21,25] is shown in Figure 2. In this case, welded carpentry modules were interlocked with a system of profiles and metal inserts (Figure 3), also in welded carpentry, and the union was stabilized by structural adhesive bonding.

Even if the modules, profiles, and inserts are welded off the assembly line, and therefore, their assembly does not preclude the simultaneous carrying out of other operations, welding still involves long processing times and thermal distortions to be restored before assembly. A driver of this study is therefore to limit the use of welding as much as possible and to simultaneously increase the modularity of the construction.

Analysis is performed starting with the “blank sheet,” following a structured methodology of product design and development [26]. Analysis of the requirements provides the fundamental features that the base has to satisfy and provides criteria for the selection of the solution concepts. The subsequent phases of the conceptual design include the functional decomposition of the base, carried out following the functional analysis [7] to derive the functional scheme of the product and the heuristic of the module [13] to collect functions in modules. From these phases follows the generation of alternative concepts, which are subsequently hierarchized according to greater or lesser correspondence to given criteria, appropriately weighted according to their importance for the purposes of the project. Finally, the selected concept is designed in detail and digitally and physically prototyped to compare it with the solution [21] and to evaluate the deformations and modal frequencies for the case of a combined filling-capping machine.

### 2.3. Requirements and Constraints Definition

The purpose of this task is to collect information about the requirements that must be fulfilled by the base, and about the existing constraints and their importance [27]. The process was carried out using a concurrent approach, where brainstorming sessions with engineers experienced in the field of beverage filling machines (thanks to a cooperation with a manufacturing company operating in this field) were performed. The requirements and constraints regarding the base to be developed can be summarized in the following points:

- Sustain suspended masses: the base must give support to the rotating parts of the machine that operate the rinsing/sanitizing and/or filling and/or capping of the beverage container.
- Transfer load to the ground: the base is stand-alone on the ground (i.e., not connected to any other structure) and transfers static and dynamic actions.
- Provide stiffness and vibration damping: the base must sustain masses and transfer loads, providing sufficient stiffness and vibration damping.
- Adaptability: the base must be adaptable to machines of all types (sterilizers, fillers, cappers, and combined groups) and sizes by composing the same items, with minimal dimensional variations of the latter.
- Cleanability: accessibility for maintenance and sanification in compliance with the regulations and directives listed below.
- Peculiar design: creating a completely new design, which differentiates the machine on the market.

Among the factors listed above, there is reference neither to cost, to favor the specifications indicated without providing any economic constraints, nor to size since the machine layout can be very different from case to case, and the new base must be modular enough to cover all the possibilities. From a regulatory point of view, the regulation and guidelines listed in Section 2.1 concerning materials are the references for the base too.

## 3. Results and Discussion

### 3.1. Functional Decomposition

**Figure 4:**
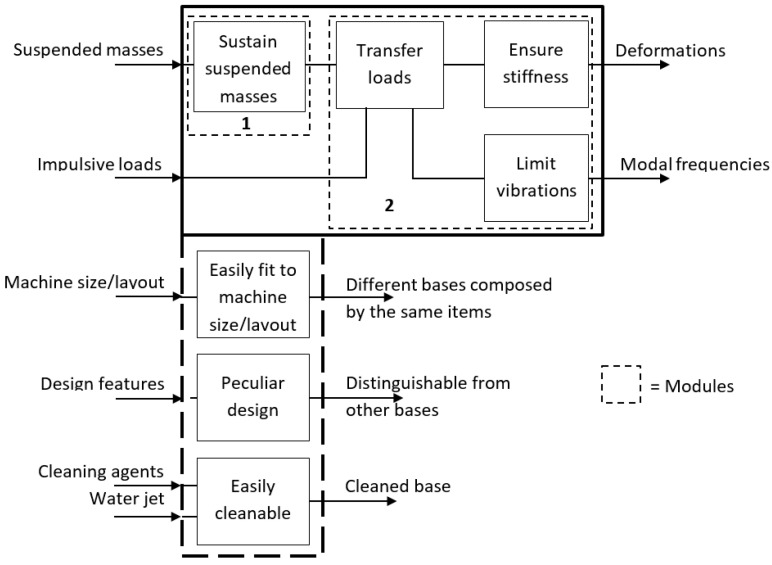
Functional decomposition of the base.

By abstracting in principle the functions that the base of an automatic machine has to perform, one can arrive at the outline depicted in the solid blackbox in Figure 4, to which are attached also the additional functions coming from the requirements and constraints described in Section 2.3 (thick dashed blackbox in Figure 4). The modules grouping the functions into physical objects are determined as those located along the dominant flow heuristic [13], which in this case practically coincides with the whole blackbox; the enclosed functions are surrounded by thin dashed blackboxes. For each module, various design options have been generated in terms of basic principles that can satisfy the enclosed functions; see the morphological matrix in Table 2. The flow that leads from the suspended masses to the deformations can be technically characterized by the stiffness parameter K (N/m), which tends to be maximized in order to minimize the deformations. The other flow, which from the impulsive loads (periodic or single) leads to the modal frequencies, is instead characterized, in the absence of further indications on the conformation of the suspended masses, by the stiffness/mass ratio of the frame itself, √K/m (1/s), which represents the fundamental frequency of a one degree of freedom vibrating system with the same stiffness and mass of the frame. The base can be built such that the fundamental frequency is above or below the production rate, which is to have a low or high K/m. The first situation can be achieved, for example, by means of a rigid but heavy monolithic structure (e.g., made by casting), while the second requires a structure optimized in stiffness/mass ratio, such as the reticular structure in Figure 2. The reticular structure can also allow the achievement of a high K, which is necessary to minimize the deformation due to the suspended masses. The function of supporting the suspended mass can be conveniently entrusted to a plate where the thrust bearing that supports the rotating part(s) of the machine can be mounted, while the function of transmitting loads and vibrations in a reticular structure is entrusted to rods.

From these considerations, a feasible combination of design options among the *n*^k^ combinations (*n* = *n*. of modules, k = *n*. of design options) is highlighted in green in Table 2, where the feasible combination is highlighted in green; that is, the base can be composed of a plate supported by a lattice structure. Parts of the rods composing the lattice are parallel to the ground and connect the structural nodes acting as ground support, while other rods are inclined with respect to the ground to transfer the loads from the plate.

**Figure 5:**
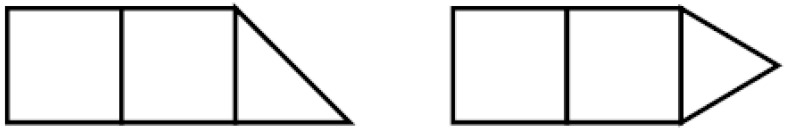
Examples of configurations of the rods parallel to the ground (upper view).

The inclined rods should be orientable in order to fit to different plate sizes and machine layouts, but it is also necessary that the rods parallel to the ground can form a polygonal structure following the machine layout (e.g., Figure 5).

Regardless of the type, the rods can be schematized as a slender element, hollow inside to minimize the mass, with suitably shaped ends to allow it to interface with the other rods in the nodes of the structure. The conceptual design problem can therefore be limited to the nodes of the structure, then defining the ends of the rods based on the type of interfacing.

### 3.2. Research of Existing Concepts

The phase after the functional decomposition is the research of already existing solutions of a structural node, both on the market and patented. The reasons for this phase are various:

- to increase the know-how regarding the product to be designed;
- to reduce the time of conceptual study thanks to the know-how acquired;
- to improve any existing solutions;
- to establish design constraints due to existing patents in order to avoid interference.

**Figure 6:**
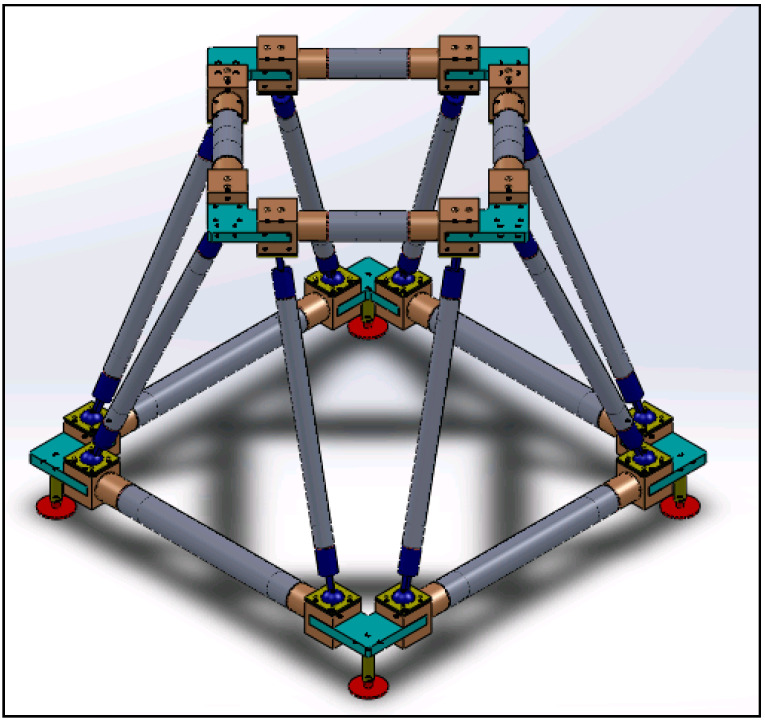
Concept of a lattice base with structural nodes alternative to the one shown in Figure 2.

Concerning already available concepts, besides the solution illustrated in Figure 2, the authors had theoretically developed a concept that replicates the lattice structure but increases the degree of modularity of the construction and that is free of welded joints. This structure, illustrated in Figure 6, consists of two square-shaped bases (the lower base is larger, and the upper one smaller, with ratios of approximately 1.4: 1) arranged on two parallel levels and rotated 45° with respect to each other, connected by eight inclined rods that give vertical support. The sides of the bases are made of tubulars (in gray) at the ends of which connecting elements (in yellow) are bonded. The latter have holes on one side for fixing to the structural nodes (colored in green), while on the opposite side they have a spherical seat for connection to the inclined rods. There is also a slot that allows the connection to engage in the structural node. The inclined rods consist of a tubular element at the ends of which are bonded elements containing a spherical pin. The locking of the latter in the seat on the yellow parts in Figure 6 takes place by means of a fastened plate. The nodes of the structure have an L shape with equal sides and are obtained from a thick sheet.

Research of patents regarding a structural node was conducted through the European Patent Office (EPO) portal [28]. In particular, joints applicable to reticular structures were selected relative, but not limited, to mechanical structures. A couple of solutions were retrieved from the market [29,30]. Other concepts were identified from different fields of application (shoulder prosthesis and pivoting wheel). A detailed list is reported in Appendix A along with a description.

### 3.3. Concept Generation of a Structural Node for an Orientable Lattice Structure

A first series of three new concepts, reported in Table 3, was inspired by the one shown in Figure 6:

- The first two exploit the components of the structure of Figure 6, but replace the L-shape structural node with a circular plate to obtain different angles between tubulars.
- The third, in addition to replacing the L-shape structural node with a plate, exploits the idea of a pivoting wheel using a double cylindrical hinge for connection with inclined rods.

A second series of new concepts, described in Table 4, on the other hand, was inspired by the patents and commercial elements referred to in Section 3.2.

### 3.4. Concept Selection

The selection of the concepts was carried out in two steps using qualitative and quantitative evaluation matrices, giving a score based on the criteria and the constraints set out in Section 2.3. The first selection matrix is presented in Table 5, where the reference concept is assigned only null scores as it is neither better nor worse than itself, while the others are evaluated as better than the reference concept (“+”), equal (“0”), or worse (“−”). The attribution of the score is done by the authors and by consulting senior designers in the field of food machines and other teachers in the machine design sector at the University of Parma, in order to have a larger evaluation panel. The emerging concepts are C, D, and F, which have the common feature of using ball joints. These concepts are passed to the quantitative evaluation phase, where the weights indicated in Table 5 are formulated in a discussion within the evaluation panel. This evaluation is quantitative in the sense that is numerical and not literal (“+” or “−”) and weighted, but at this conceptual stage, the design is not sufficiently defined to set out quantitative values for each parameter; therefore, the score is assigned based on a synthetical judgment.

The concept that obtained an intermediate score among those selected in the qualitative evaluation was taken as a reference for the quantitative selection, therefore assigning it a score of 3 for all criteria (i.e., intermediate between 1 (minimum satisfaction of the criterion) and 5 (maximum satisfaction)). The criteria were also reduced to four since flexibility and modularity were equally met by all selected concepts in the qualitative assessment. The results are summarized in Table 6, where the F concept is the best performing, albeit of narrow measure.

From the analysis of Table 6, it can be noted that the small difference in score between concepts F and D is mainly due to the manufacturing criterion, which has a low weight but in which F scores less. The reason is that the F concept contains numerous parts, for which a precision machining is also required. On the other hand, the D concept is more easily fabricated mainly due to the smaller number of parts, but it requires more effort in the precise positioning and in the locking of the ball joints during the assembly phase, where it has been assigned a lower score. During the detail design phase, therefore, an attempt will be made to merge the positive aspects of concepts D and F to try to eliminate or diminish the negative ones.

### 3.5. Detail Design

**Figure 7:**
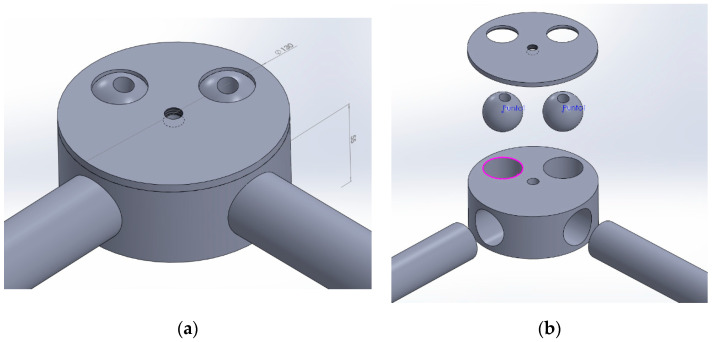
Structural node version 1: assembly view (**a**); exploded view (**b**).

To improve manufacturability compared with concept F and at the same time to decrease the number of ball joints compared with concept D, a structural node was designed consisting of two elements instead of four as in F and with bonded cylindrical joints for the horizontal rods instead of mechanically fixed spherical ones. This solution is represented in Figure 7.

**Figure 8:**
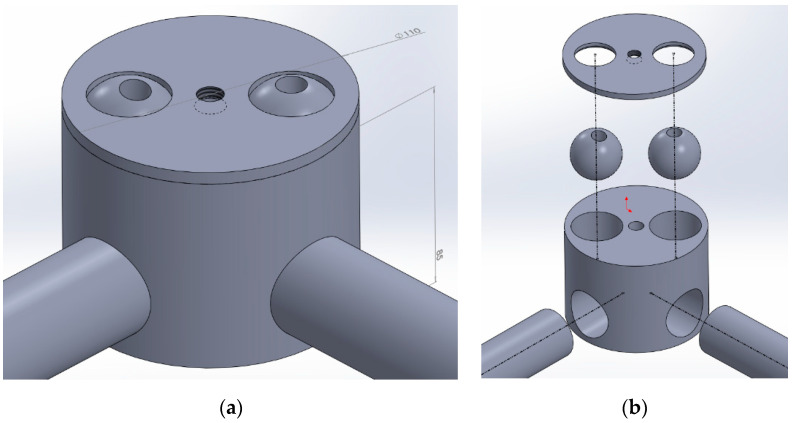
Structural node version 2: assembly view (**a**); exploded view (**b**).

This configuration of the node leads to having the spherical joints of the inclined rods off-center with respect to the axis in which the horizontal rods converge. This induces a moment on the node due to the loads coming from the inclined rods, a moment that would be good to eliminate or to minimize in order to have less stress and deformation of the node itself and therefore of the frame. To this end, a second solution has been developed in which the seats for the spheres are offset in height with respect to the horizontal rods, and it is therefore possible to keep their axes in a plane that contains the axis of the node (Figure 8).

The dimensional tolerances assigned to the depth of the holes containing the balls and to the holes on the cover that enclose them are such so as to always generate a size interference that stabilizes the connection once the cover is tightened. The gap that forms between the sphere and the cover on the outside must then be suitably leveled and sealed with resin to prevent the accumulation of residues that are difficult to remove during the periodic washing and sanitization of the machine. The realization of angles between the horizontal rods different from 90° illustrated in Figure 8 obviously requires the creation of ad hoc holes. This means loosing a minimum of modularity compared with the original concepts D and F, but still gaining on the simplification of manufacturing and assembly. The components necessary for the construction of the structural node illustrated in Figure 8 are obtained by cutting and machining, except the balls, which are commercial and equipped with a threaded hole in which to insert a stud. The rods are equipped with bonded, threaded terminals with a right/left thread at the two opposite sides. This allows for connecting to the spherical hinges with a fine adjustment to ensure the horizontality of the support plate of the rotating part of the machine.

The assembly cycle of the base, first of all, involves the adhesive bonding of tubes inside the holes of the structural nodes in order to create a subassembly. Once the adhesive has cured, the rods with bonded, threaded terminals are screwed onto the studs on spherical hinges. Structural nodes are placed at the upper side of the rod to support the plate, which is the last part to be positioned. After the fine adjustment of the position of the rods and their tightening, the support plate is bonded to the upper nodes.

### 3.6. Virtual and Physical Prototyping

A first analysis compared the base [21] with an equivalent version of the new base (FLEX), where rods are tubular profiles with a 40 mm external diameter and a 2 mm wall thickness. The simulation was conducted with the finite element method using the simulation module of the SolidWorks software package, applying the weight of the carousel and of the other devices to a reference point along the axis of the machine.

**Figure 9:**
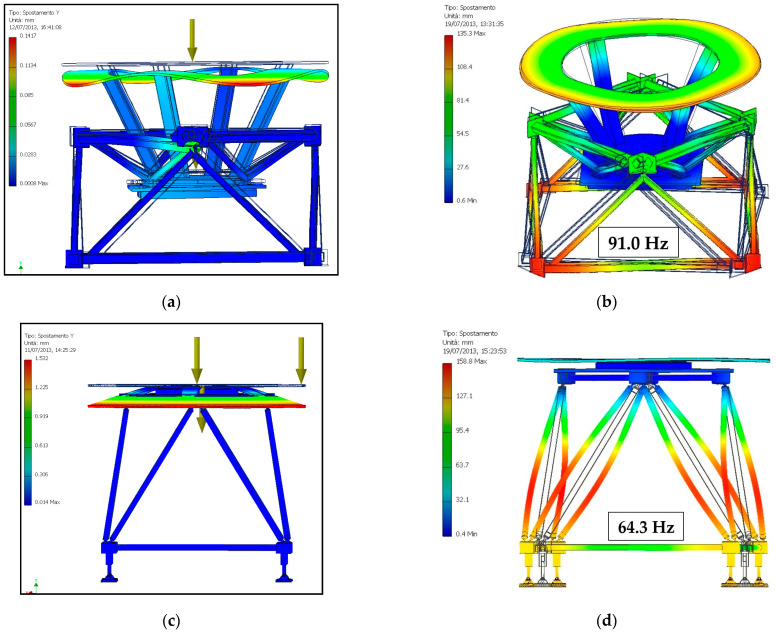
Displacement (**a**) and first vibration mode (**b**) of the base [21]; displacement (**c**) and vibration mode (**d**) of the corresponding FLEX base.

It should be noted that the two carousels are not identical, but that of the FLEX base is a “disk” with a simpler geometry than the turret one mounted on the base developed in [21]. A vertical translation constraint is imposed on all the supports, while one is fully constrained in order to avoid rigid body motions. Relative movement between the bodies in contact is also prevented. The stress in the various components never reaches levels of attention, so only the results relating to the deformation and the first modal frequency due to the frame are reported (the first frequencies are related to the carousel). In Figure 9, the displacements of the base developed in [21] and the FLEX base are compared, and the first modal frequencies are represented.

It is also noted that in both cases, the deformation is more localized in the carousel, with very low values relating to the base ranging from 0.03 mm of the base [21] to 0.02 mm of the corresponding FLEX base. This difference is at least partly due to the greater weight of the turret of the base of [21]. Regarding the modal analysis, the base [21] reveals a first vibration mode at 91 Hz versus the 64.3 Hz of the FLEX base, which is more slender. However, both of these values are largely higher than the forcing frequency of a bottling system, corresponding to the number of bottles processed per second, which, for medium-sized machines, remains below 10 Hz.

**Figure 10:**
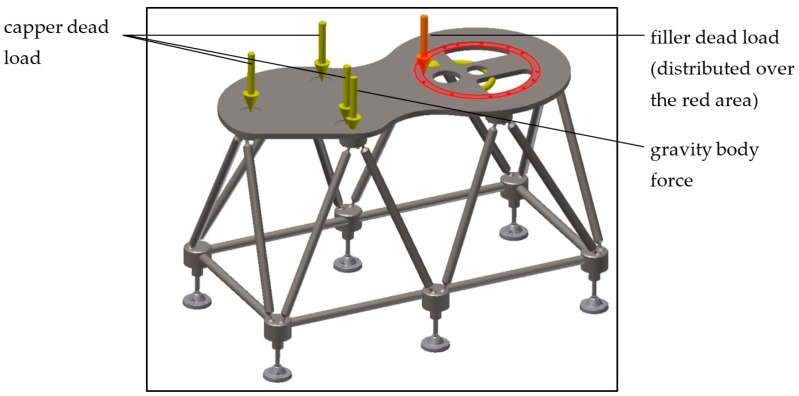
FLEX base model for a filler–capper combined unit.

Given the substantial equivalence of the FLEX base to the base [21] for a corresponding machine size, the simulation moved towards that of a larger FLEX base, designed for a combined filler–capper group. The size of this base is approximately 1600 × 800 × 1000 mm^3^ (length × width × height). In this case, only the base was modeled to computationally lighten the solution by applying gravity to the modeled parts and distributing the weight forces in correspondence with the area where the thrust bearing (filler) is present and in correspondence with the three supports (capper) (Figure 10). The constraints always include one support completely restrained and the others with vertical restraints.

**Figure 11:**
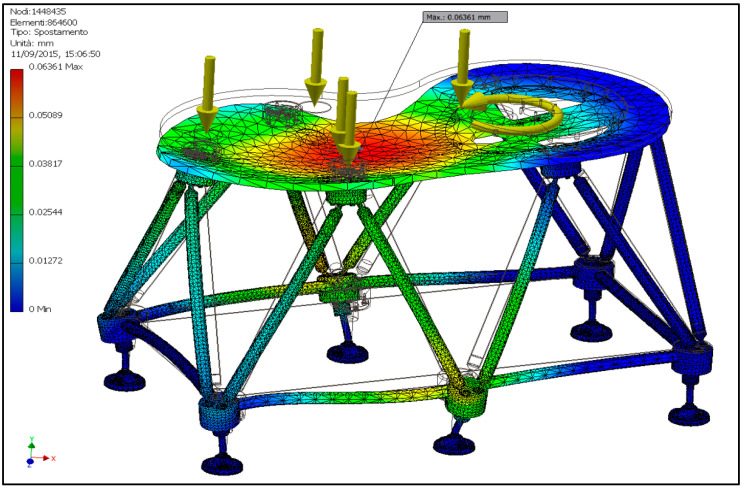
Displacements of the FLEX base for a filler–capper combined unit.

**Figure 12:**
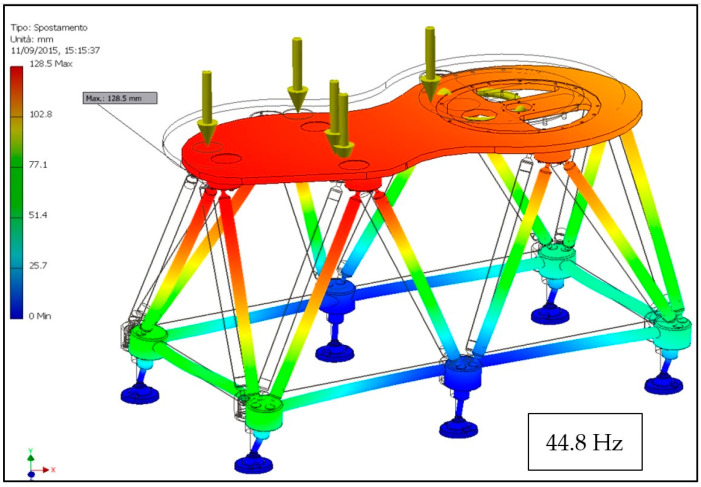
First mode of vibration of the FLEX base for a filler–capper combined unit.

The results of the analysis in terms of displacements and natural frequency are shown in Figure 11 and Figure 12, respectively, but also in this case, the tensions are not reported as they are modest. It is immediately evident how, in this case, even though the structure is larger than the one analyzed previously, the displacements are contained within 0.06 mm (part of them attributable to flexural deformation of the support plate), a value that is absolutely compatible with the good functioning of the machine. Regarding the modal analysis, the first natural frequency occurs at 44.8 Hz, lower than that shown Figure 9d as expected by virtue of the larger size but still well above the forcing frequency of these machines.

**Figure 13:**
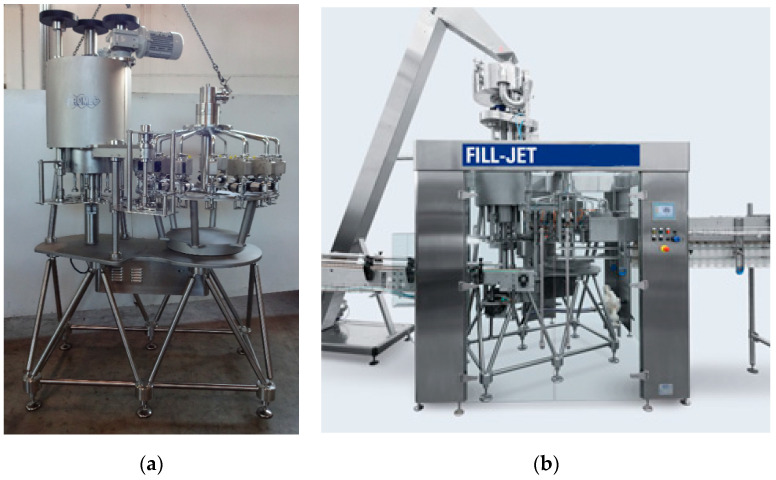
Prototype of the combined filler–capper unit with a FLEX base: (**a**) without and (**b**) with external protections.

From the analyses conducted, the overall result is that there are technological and performance conditions for the construction of a physical prototype, whose practical implementation is shown in Figure 13. The prototype has passed the no-load test and has been placed on trial at a company, where it is still active 5 years since its commissioning, that witnesses that the design and development process adopted has led to an innovative and reliable product.

## 4. Conclusions

The theme of the conception and development of a configurable lattice base, which would limit the use of welding as much as possible and at the same time would increase the modularity of the construction, was tackled starting with the “blank sheet” following a structured methodology of product design and development. The result is a modular and adaptable base (FLEX base), which has been digitally prototyped to compare it with solutions previously developed by some of the authors, revealing comparable characteristics of stiffness and first modal frequency. Finally, a version was manufactured for a filler–capper combined machine, where it has been in operation at a company for 5 years without showing any problems, demonstrating that the development process adopted has achieved the innovation objectives set while ensuring the durability of the product.

## Figures and Tables

**Table 1:** Loctite EA 9466 mechanical properties (in MPa).

Lap Shear Strength *	Tensile Strength **	Young’s Modulus
23	32	1718

* on stainless steel; ** bulk adhesive.

**Table 2:** Morphological matrix for the construction of different concepts of the base.

Module	Design Option 1	Design Option 2	Design Option 3
Module 1	Plate	Lattice	Skin + stringers
Module 2	Monobloc	Lattice	Monocoque

**Table 3:** Concepts conceived starting with the structure shown in Figure 6.

Concept Name	Concept Sketch	Concept Description
(**A**)	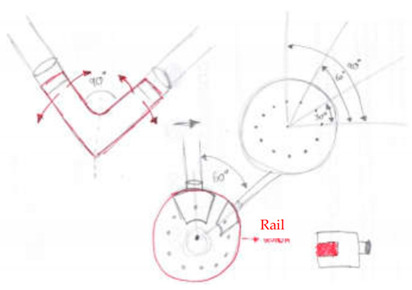	Joint consisting of a perforated circular base that acts as a guide for two rotating sliders. Once the sliders have reached the positions to form the desired opening angle, they are fixed, each with two pins/fasteners, to the base of the node.
(**B**)	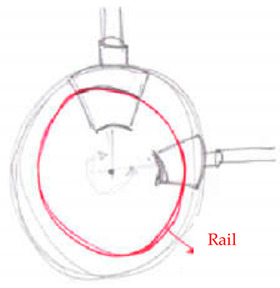	Evolution of the previous solution. The circular base is no longer perforated, but equipped with a rail that is also circular, on which two cursors can rotate to reach the desired opening angle. The fastening takes place by means of pins, which bind the sliders to the guide, which is drilled in the radial direction.
(**C**)	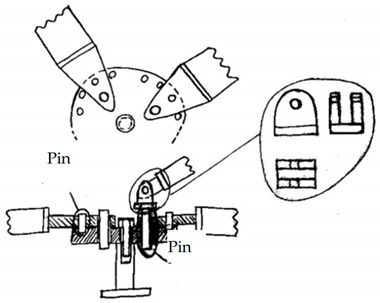	The solution provides a base plate on which two further plates are fixed by means of pins and screws, connected in turn to the rods. An additional element is fixed to the pin to hook the inclined rods. This last solution for hooking the inclined rods can be easily extended to concepts A and B.

**Table 4:** Concepts devised starting with the patents and commercial elements referred to in Section 3.2.

Concept Name	Concept Sketch	Concept Description
(**A**)	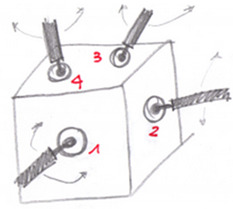	The solution provides a cube on which seats are manufactured to host several ball joints, which allow any shape of the base of the structure and inclination of the rods. The ball joint is fixed as in Figure 6 by a flange fastened to the cube and/or bonded with a structural adhesive.
(**B**)	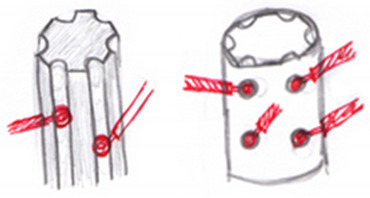	It consists of a cylindrical block with semicircular grooves where spheres (in red) are positioned. The locking of the spheres on the groove takes place by means of a casing. The casing has also the function of sealing the inside and making the joint more hygienic. The ring is closed at the ends by two plates. With respect to the patent PR2013A000019 shown in Appendix A, the spheres are kept in position by adhesive bonding or welding.
(**C**)	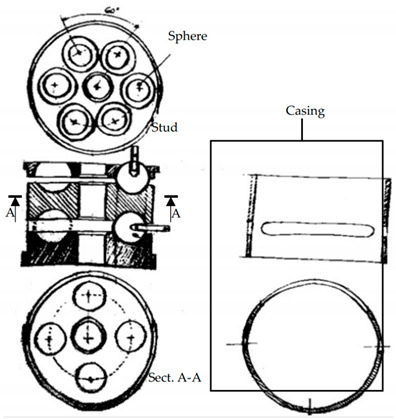	It consists of three overlapping machined plates with hemispherical seats for commercial spheres with a threaded hole to insert a stud. The plates have cavities that accommodate a sphere for connection with both the horizontal and the inclined rods. The entire system of plates and spheres is enclosed within a casing that seals the node.

**Table 5:** Qualitative evaluation of the concepts (better (“+”), equal (“0”), or worse (“−”) than the reference concept).

Criterion	Ref. Concept (Figure 6)	A	B	C	D	E	F
Adaptability	0	+	+	+	+	+	+
Modularity	0	0	0	0	0	0	0
Cleanability	0	0	0	0	+	+	+
Design	0	0	0	0	0	+	+
Manufacturing	0	0	0	0	+	−	−
Assembly	0	0	0	+	−	−	+
Total +	0	1	1	2	3	3	4
Total 0	5	5	5	4	2	1	1
Total −	0	0	0	0	1	2	−
Sum	0	1	1	2	2	1	3
Ranking	6	4	4	2	3	5	1

**Table 6:** Quantitative evaluation of the three concepts with the highest scores in Table 5.

Criterion	Weight	C	D	F
Cleanability	0.3	3	4	4
Design	0.1	3	4	5
Manufacturing	0.2	3	4	2
Assembly	0.4	3	2	3
Sum	1	3	3.1	3.2
Ranking	-	3	2	1

**Figure A1:**
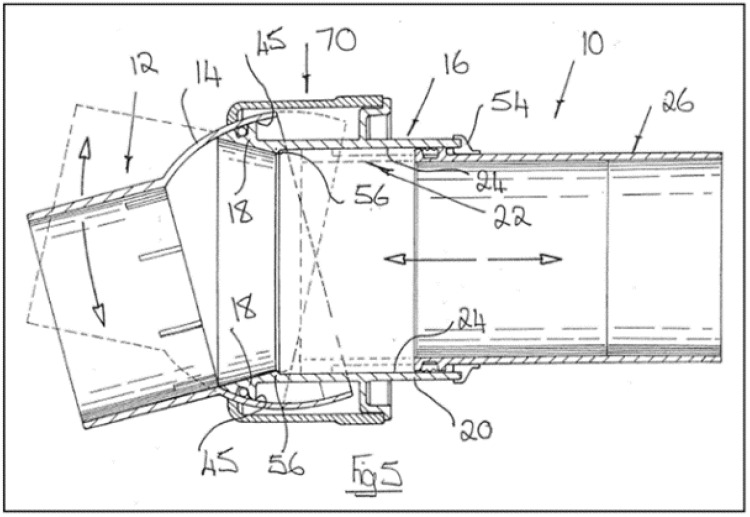
Scheme of the joint for pipelines, patent AU2011101376A4.

**Figure A2:**
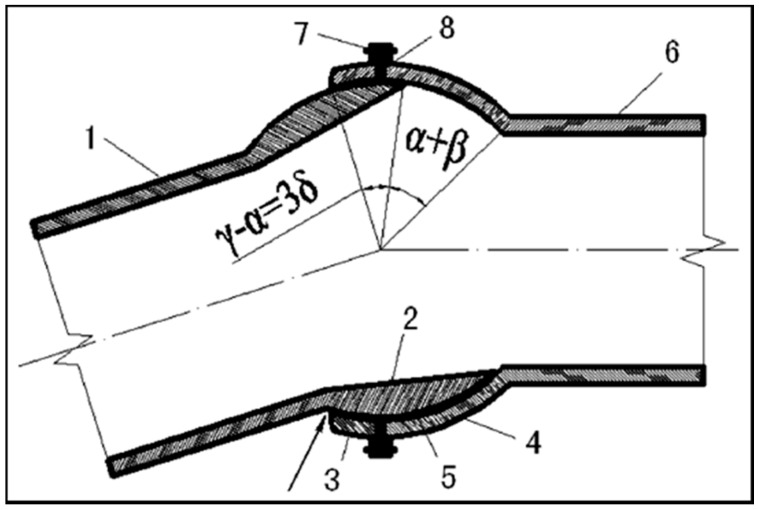
Scheme of the joint for pipelines, patent CN102588689A.

**Figure A3:**
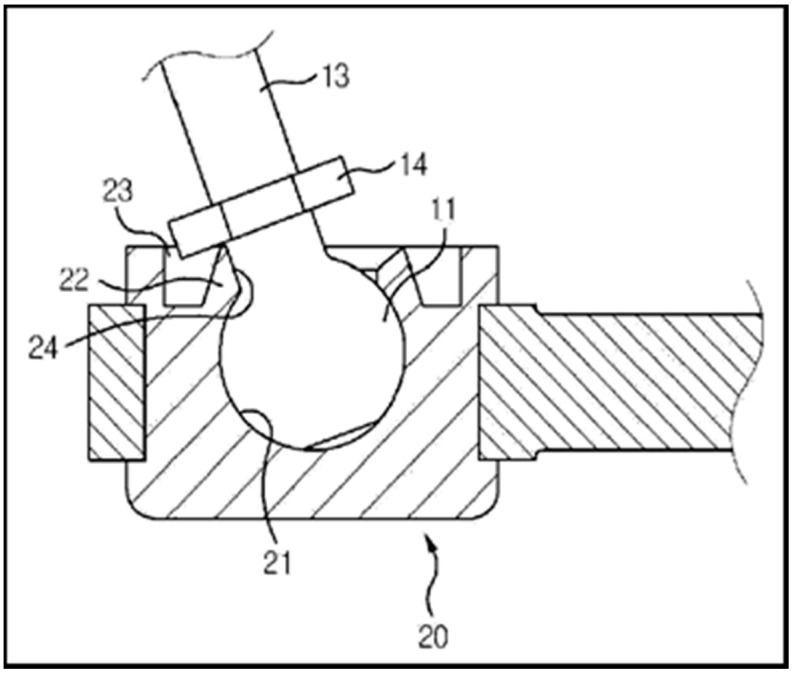
Scheme of the ball joint, patent EP2514982A2.

**Figure A4:**
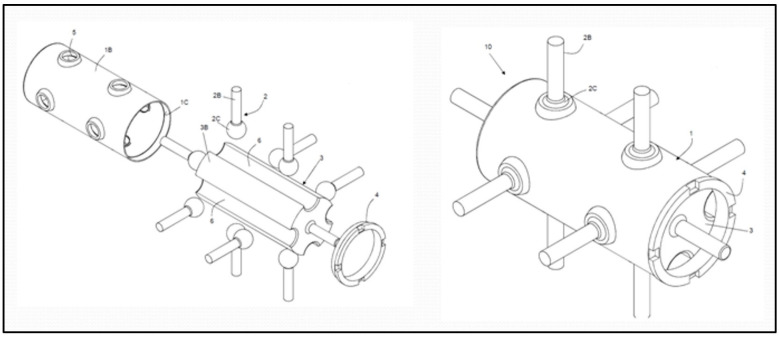
Scheme of the universal structural node, patent PR2013A000019.

**Figure A5:**
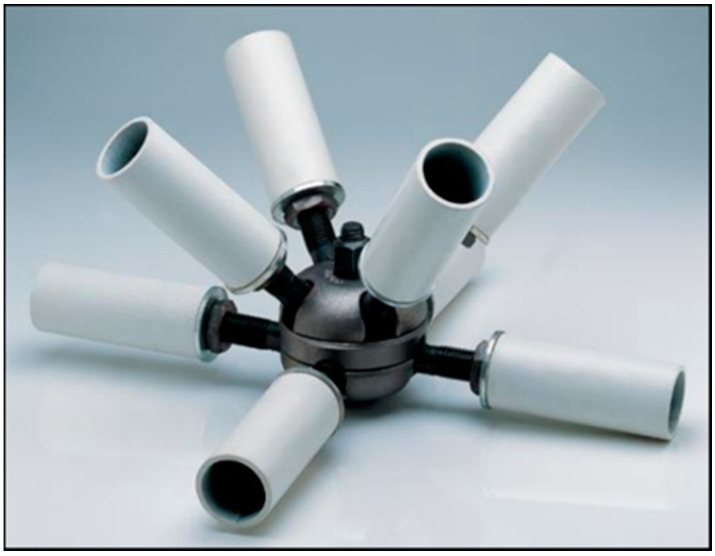
Vestrut node, from [29].

**Figure A6:**
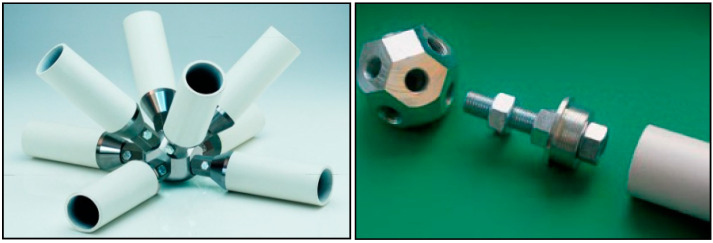
Cubotto node, from [30].

## Data Availability

The data presented in this study are available on request from the corresponding author.

## Appendix A

Swivel Joint Assembly for Coupling Two Conduits to Each Other (AU2011101376A4)

The joint in Figure A1 is generally used for the coupling of two tubular pipes incident to each other. It is essentially composed of three parts: a tube with a truncated spherical end facing outwards, a tube with a truncated conical end that is coupled to the inside of the previous one, and a sleeve that allows the two tubes to be tightened.

It is interesting for the purposes of the base as it would allow for making this structure modular, with the advantage of being able to adapt it to the different needs of the customer simply by varying the length of the tubes, without necessarily having to redesign the entire structure.

Pipeline Turning Joint (CN102588689A)

This is very similar to the previous one, but it is simpler (Figure A2). At the ends of the two tubes, two spherical surfaces are obtained, which couple one inside the other. Two screws secure the two tubes in the pre-established position.

Ball Joint (EP2514982A2)

It is a joint with a very simple structure (Figure A3) composed of a tube with a spherical end, which is coupled with a body with a spherical seat. The joint allows three rotational degrees of freedom, but it is necessary to provide a locking system to fix the relative position of the tubes.

This type of joint is also available with a protection that allows for the isolation of the joint from dust and with a component that could allow for the locking of the ball inside the seat.

Universal Structural Node (PR2013A000019)

The main feature of this connection element (Figure A4) is having a certain number of couplings (greater than two) where rods can be welded, bonded, or screwed, with a variable relative orientation in such a way as to coincide with that of the rods of the structure. In this way, it is possible to connect a multiplicity of rods with relative orientations that can vary as desired within a certain range.

Vestrut [29]

This is a joint characterized by the presence of several spherical hinges in hot-pressed, hardened, and tempered steel and tubular rods with a circular section in low-alloy steel. The joint consists of three circular elements, two caps and a central plate that encloses them, joined together by a single screw: the two symmetrical caps are equipped with slots and housings in which the rod ends are positioned during assembly (Figure A5).

The spherical head terminals are fixed by a lock nut and screwed directly onto the tapered rods, right-handed on one side and left-handed on the other. Welds are excluded for making all connections between the system components and simultaneous screwing in the node with up to 12 rods.

Cubotto [30]

This joint system is composed of tubular rods with a stud terminal that connects to a threaded polygonal metal block (Figure A6). It is obtained entirely by mechanical processing on numerically controlled machines, so it is possible to create any type of three-dimensional reticular geometry with a tetrahedral and/or semi-octahedral module regardless of lengths and loads. The connections between the various components of the system (rods and nodes) are made without the use of welding.

Shoulder Prosthesis

The shoulder prosthesis is a spherical hinge that would allow the orientation of the rods of a frame in any direction. The prosthesis is available both in the classic version, with the head placed at the end of the stem terminating the humerus, and in the reverse one, with the hemispherical head placed directly on the shoulder bones and the cap seat grafted onto the terminal part of the humerus.

Pivoting wheel

A device such as a common trolley wheel allows two elements to be connected, allowing them the possibility of relative rotation. The wheel pin constitutes a cylindrical hinge, which, together with the other degrees of freedom, allows a spatial orientation like a spherical joint.
